# HIV‐free survival at 12–24 months in breastfed infants of HIV‐infected women on antiretroviral treatment

**DOI:** 10.1111/tmi.12710

**Published:** 2016-05-24

**Authors:** Lana Clara Chikhungu, Stephanie Bispo, Nigel Rollins, Nandi Siegfried, Marie‐Louise Newell

**Affiliations:** ^1^ School of Languages and Area StudiesUniversity of PortsmouthPortsmouthUK; ^2^ Department of Social Statistics and DemographyUniversity of SouthamptonSouthamptonUK; ^3^ Department of Maternal, New‐born, Child and Adolescent HealthWorld Health OrganizationGenevaSwitzerland; ^4^ Independent Clinical Epidemiologist; ^5^ Human Development and HealthFaculty of MedicineUniversity of SouthamptonSouthamptonUK

**Keywords:** HIV‐free survival, antiretroviral treatment, women, systematic review, survie sans VIH, ART, femmes, revue systématique

## Abstract

**Objective:**

To provide estimates of HIV‐free survival at 12–24 months in breastfed children by maternal ART (6 months or lifelong) to inform WHO HIV and Infant Feeding guidelines.

**Methods:**

Eighteen studies published 2005–2015 were included in a systematic literature review (1295 papers identified, 156 abstracts screened, 55 full texts); papers were analysed by narrative synthesis and meta‐analysis of HIV‐free survival by maternal ART regimen in a random effects model. We also grouped studies by feeding modality. Study quality was assessed using a modified Newcastle–Ottawa Scale (NOS) and GRADE.

**Results:**

The pooled estimates for 12‐month HIV‐free survival were 89.8% (95% confidence interval, CI: 86.5%, 93.2%) for infants of mothers on ART for 6 months post‐natally (six studies) and 91.4% (95% CI 87.5%, 95.4%) for infants of mothers on lifelong ART (three studies). Eighteen‐month HIV‐free survival estimates were 89.0% (95% CI 83.9%, 94.2%) with 6 months ART (five studies) and 96.1% (95% CI 92.8%, 99.0%) with lifelong ART (three studies). Twenty‐four‐month HIV‐free survival for infants whose mothers were on ART to 6 months post‐natally (two studies) was 89.2% (95% CI 79.9%, 98.5%). Heterogeneity was considerable throughout. In four studies, HIV‐free survival in breastfed infants ranged from 87% (95% CI 78%, 92%) to 96% (95% CI 91%, 98%) and in formula‐fed infants from 67% (95% CI 35.5%, 87.9%) to 97.6% (95% CI 93.0%, 98.2%).

**Conclusion:**

Our results highlight the importance of breastfeeding for infant survival and of ART in reducing the risk of mother‐to‐child HIV transmission and support the WHO recommendation to initiate ART for life immediately after HIV diagnosis.

## Abstract (fr)

**Objectif:**

Fournir des estimations sur la survie sans VIH entre 12 et 24 mois chez les enfants allaités par des mères sous ART (durant 6 mois ou pour toujours), afin d'informer les directives de l’OMS sur le VIH et l'alimentation des nourrissons.

**Méthodes:**

Dix‐huit études publiées entre 2005 et 2015 ont été incluses dans une analyse systématique de la littérature (1295 articles identifiés, 156 résumés screenés et 55 textes complets); les articles ont été analysés par synthèse narrative et une méta‐analyse de la survie sans VIH sous un régime ART de la mère dans un modèle à effets aléatoires. Nous avons également regroupé les études par mode d'alimentation. La qualité des études a été évaluée à l'aide de l'Echelle de Newcastle‐Ottawa (NOS) et de GRADE.

**Résultats:**

Les estimations poolées pour de la survie à 12 mois sans VIH étaient de 89,8% (intervalle de confiance IC à 95%: 86,5% ‐ 93,2%) pour les nourrissons de mères sous ART durant 6 mois après la naissance (six études) et de 91,4% (IC95%: 87,5% ‐ 95,4%) pour les nourrissons de mères sous ART pour toujours (trois études). Les estimations de la survie à 18 mois sans VIH étaient de 89,0% (IC95%: 83,9% ‐ 94,2%) durant 6 mois sous ART (cinq études) et de 96,1% (IC95%: 92,8% ‐ 99,0%) sous ART pour toujours (trois études). La survie à 24 mois sans VIH pour les nourrissons dont les mères étaient sous ART durant 6 mois après la naissance (deux études) était de 89,2% (IC95%: 79,9% ‐ 98,5%). L'hétérogénéité était considérable tout au long du suivi. Dans quatre études, la survie sans VIH chez les nourrissons allaités au sein variait de 87% (IC95%: 78% ‐ 92%) à 96% (IC95%: 91% ‐ 98%) et chez les nourrissons nourris au lait de formulation, de 67% (IC95%: 35,5% ‐ 87,9%) à 97,6% (IC95%: 93,0% ‐ 98,2%).

**Conclusion:**

Nos résultats soulignent l'importance de l'allaitement maternel pour la survie du nourrisson et de l’ART dans la réduction du risque de transmission du VIH de la mère à l'enfant et soutiennent la recommandation de l’OMS d'initier l’ART pour la vie, immédiatement après le diagnostic du VIH.

## Abstract (es)

**Objetivo:**

Proveer cálculos sobre supervivencia a los 12‐24 meses, libre de VIH, de niños recibiendo lactancia materna de madres en TAR (6 meses o de por vida) que sirvan a las directrices de la OMS sobre VIH y alimentación.

**Métodos:**

Se incluyeron dieciocho estudios publicados entre 2005‐2015, obtenidos mediante una revisión sistemática de la literatura (1295 artículos identificados, 156 resúmenes evaluados, 55 textos completos leídos); los artículos se analizaron mediante una síntesis narrativa y meta‐análisis de supervivencia libre de VIH según el régimen materno de TAR en un modelo de efectos aleatorios. También agrupamos estudios según la modalidad de alimentación. La calidad del estudio se evaluó utilizando una modificación de la Escala Newcastle‐Ottawa (ENO) y GRADE.

**Resultados:**

Los cálculos de supervivencia sin VIH agrupados a los 12 meses eran del 89.8% (intervalo de confianza (IC) 95%: 86.5%, 93.2%) para bebés de madres recibiendo TAR durante 6 meses después del parto (seis estudios) y 91.4% (IC 95% 87.5%, 95.4%) para bebes de madres recibiendo TAR de por vida (tres estudios). Los datos de supervivencia sin VIH a los 18 meses eran del 89.0% (IC 95% 83.9%, 94.2%) con 6 meses de TAR (cinco estudios) y 96.1% (IC 95% 92.8%, 99.0%) con TAR de por vida (tres estudios). La supervivencia sin VIH a los 24‐meses para niños cuyas madres estaban recibiendo dar hasta 6 meses después del parto (dos estudios) era del 89.2% (IC 95% 79.9%, 98.5%). La heterogeneidad era considerable. En cuatro estudios, la supervivencia sin VIH en bebes que recibieron lactancia materna estaba entre el 87% (IC 95% 78%, 92%) y el 96% (IC 95% 91%, 98%) y en bebés alimentados con leche de fórmula entre el 67% (IC 95% 35.5%, 87.9%) y el 97.6% (IC 95% 93.0%, 98.2%).

**Conclusión:**

Nuestros resultados subrayan la importancia de la lactancia para la supervivencia infantil y del TAR en reducir el riesgo de la transmisión madre‐hijo del VIH, y apoyan la recomendación de la OMS de iniciar TAR para toda la vida inmediatamente después del diagnóstico del VIH.

## Introduction

The 2010 WHO infant feeding guidelines in the context of HIV infection recommended exclusive breastfeeding (EBF) for 6 months followed by complementary feeding and continued breastfeeding (CBF) for up to 1 year, under the cover of antiretroviral treatment (ART) to either the mother or the infant 1. However, these recommendations were based on limited evidence in terms of final HIV‐free survival and also toxicity of ART to infants where the infant received prophylaxis to prevent mother‐to‐child transmission (PMTCT). Since then, further evidence has become available from studies and programmes where PMTCT post‐natally was achieved through maternal ART or infant ARV prophylaxis, and a summary of these data could provide information for recommendations on duration of breastfeeding.

In late 2015, WHO revised HIV ART guidelines to include treatment for all immediately upon diagnosis of HIV infection 2. This systematic review was commissioned by WHO and contributed to the development of the 2015 WHO HIV Infant Feeding guidelines. We present the results from a systematic review and the associated GRADE evidence summary tables of HIV‐free survival at 12, 18 and 24 months in infants born to women on ART for life or for 6 months post‐partum and by infant feeding modality.

## Methods

Our systematic review considered both experimental and observational studies and included HIV‐positive mothers receiving ART and their breastfed children. Infants may also have received prophylactic ART as per WHO 2010 guidelines. Exposure was defined as HIV ART (and duration) and breastfeeding (and duration), and outcome measures were HIV‐free survival and overall and post‐partum HIV transmission between birth and 24 months of age.

**Figure 1:**
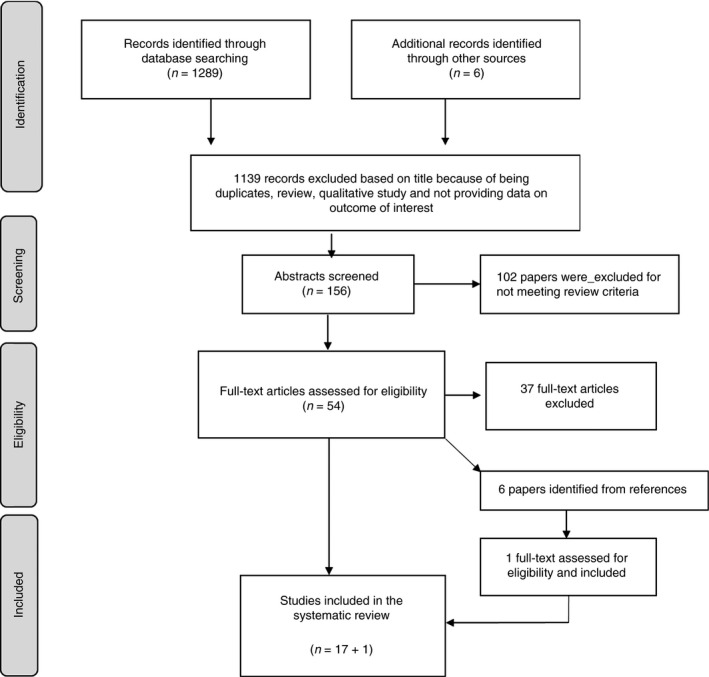
Flowchart of screening process. Search Terms in PubMed (((Maternal[Title/Abstract] OR mother*[Title/Abstract]) AND (Antiretroviral therapy[Title/Abstract] OR Antiretroviral*[Title/Abstract] OR ART[Title/Abstract] OR ARV[Title/Abstract] OR HAART[Title/Abstract]) AND (HIV free survival[Title/Abstract] OR HIV[Title/Abstract] OR Transmi*[Title/Abstract] OR Death[Title/Abstract] OR Mortality[Title/Abstract]) AND (Breastfeeding[Title/Abstract] OR Postnatal[Title/Abstract] OR Breast*[Title/Abstract])) AND (“2005”[Publication Date] : “2015”[Publication Date])).

SB, LB and MLN searched English literature from multiple electronic databases including PubMed, MEDLINE, EMBASE, Cochrane Central Register of Controlled Trials, Web of Science and CINAHL for articles published between 2005 and 2015. The search words in PubMed are shown in a footnote to Figure 1. The search terms were adapted for other databases.

Reference lists from relevant studies, grey literature and online abstracts from the International IAS AIDS Conference in Melbourne 2014 and the 2013–2015 Conferences on Retroviruses and Opportunistic Infections were also searched. Reference lists of articles identified from the search of databases and conference abstracts were scrutinised, and in cases where relevant information was not available in the publication, authors were contacted for specific additional information regarding infant feeding modality.

The quality of studies included in the analysis was assessed using a modified Newcastle–Ottawa Scale (NOS) 3, 4 (Table S1). Although some of the included studies were nested within randomised controlled trials, randomisation was not based on the intervention of interest (breastfeeding) and these studies were considered cohorts. Each study could score a maximum of six stars on selection and four on outcome; factors considered included the representativeness of the study population, ascertainment of exposure to ART and breastfeeding, initiation on basis of ART eligibility, maternal adherence to ART and duration of breastfeeding. Ascertainment of outcome (HIV‐free survival) included timing of assessment and whether the outcome was stratified by feeding, length of follow‐up and loss to follow‐up.

The information obtained from the NOS was used to comment on the quality of the included studies in GRADE with respect to study limitations/risk of bias 5. We also considered consistency of results, directness, precision and publication bias.

### Synthesis of evidence

We undertook a narrative synthesis and obtained pooled estimates of HIV‐free survival with a heterogeneity score based on a random effects meta‐analysis in STATA 13 (Stata Corp, 2013). We also summarised the information in graphs depicting HIV‐free survival rates by duration of maternal ART where possible and additionally presented HIV transmission rates for studies that provided transmission rates at 6 months and at the end of follow‐up. If no confidence interval for HIV‐free survival estimated was provided, it was calculated based on the number of events and those at risk using the formula described by Eayres 6.

## Results

The search process identified 1295 citations, of which 1139 were excluded on the basis of being a duplicate, review, qualitative study or not evaluating transmission, mortality or HIV‐free survival (Figure 1). Abstracts of the remaining 156 studies were evaluated by SB and LC, and 54 texts were selected for full screening. SB, LC and MLN undertook the full‐text screening. Six additional articles from the references were identified through full‐text screening, bringing the total to 60 (Figure 1). Eighteen studies were included in the analysis. Details of excluded studies with reason are provided in Table S2. Eight additional papers from selected studies provided additional information for the assessment of quality of studies and data collection 7, 8, 9, 10, 11, 12, 13, 14.

We present the evidence using a narrative synthesis, in addition to providing a pooled estimate with a heterogeneity score based on a random effects meta‐analysis, as recommended for use in the analysis of studies of different design 15. Of the 18 selected studies, one was performed in India and the remaining 17 were from African countries. Five studies were conducted in rural areas 16, 17, 18, 19, 20 and the remainder in urban areas. All 18 were cohort studies, of which seven were nested within randomised clinical trials (see Table S3 for full study details). Most studies were a follow‐up of mothers receiving ART for prevention of mother‐to‐child transmission (PMTCT) 16, 18, 19, 21, 22, 23, 24, 25, with mothers advised to exclusively breastfeed for 6 months with rapid weaning thereafter, in line with the prevailing WHO recommendations. Seven studies offered lifelong ART irrespective of CD4 count and supported breastfeeding for 12 months 16, 21, 22, 25, 26, 27, 28. Thomas *et al*. 29 presented findings from a clinical trial evaluating two combination ART regimen, which was included as a cohort of all women on ART post‐natally. Thakwalakwa *et al*. 16 randomised mothers/infants post‐partum to two types of complementary foods after weaning; all mothers received ART lifelong including throughout the breastfeeding period. Tonwe‐Gold *et al*. 25 presented findings of a cohort of women who received ART for life if they were ART‐eligible as per prevailing WHO guidelines or who received short‐course ART for PMTCT only; data are included accordingly.

The seven cohorts nested within clinical trials evaluated the use of ART during pregnancy and post‐natally in reducing mother‐to‐child transmission (MTCT) of HIV and infant deaths 8, 16, 28, 29, 30, 31, 32. Cournil *et al*. 8 in the Kesho Bora study compared ART through 6 months of breastfeeding with short duration of peripartum ART and presented HIV‐free survival in children who were never breastfed and those who were breastfed for more than 3 months. Jamieson *et al*. 32 and Coovadia *et al*. 30 compared rates and risk by prolonged infant nevirapine prophylaxis, with mothers on different types of ART, but provided separate estimates on HIV‐free survival for the group of mothers on ART for at least 6 months.

### Quality of studies

Table S4 presents findings of the assessment of the quality of the studies based on the modified Newcastle–Ottawa Scale. The study by Ngoma *et al*. 27 had the highest quality in terms of selection (six stars), followed by Sagay *et al. et al*. 26, Cohan *et al*. 28, Jamieson *et al*. 32, Thomas *et al*. 29 and Peltier *et al*. 20, all with 4 stars. The studies by Alvarez‐Uria *et al*. 17 and Tonwe‐Gold *et al*. 25 scored highest on quality of outcome assessment (4 stars).

Most studies did not provide details on type of feeding and assumed (but did not formally document) that most mothers exclusively breastfed up to 5 or 6 months as recommended. Estimates of HIV‐free survival by type of feeding could only be obtained from four studies 8, 17, 19, 20. Tonwe‐Gold 25 presented information on HIV transmission by feeding type and Cournil *et al*. 8 presented rates in children who were either formula‐fed from birth, breastfed for less than 3 months or for 3 months or longer. Three studies compared transmission or death between breastfed and formula‐fed infants 17, 19, 20.

Eleven studies provided HIV‐free survival or rates of transmission and mortality from birth 18, 19, 23, 24, 25, 26, 27, 28, 29, 31, 32. Other studies excluded deaths and HIV transmission in the first days or weeks of life and provided only post‐natal rates.

### HIV‐free survival

#### At age 12 months

**Figure 2:**
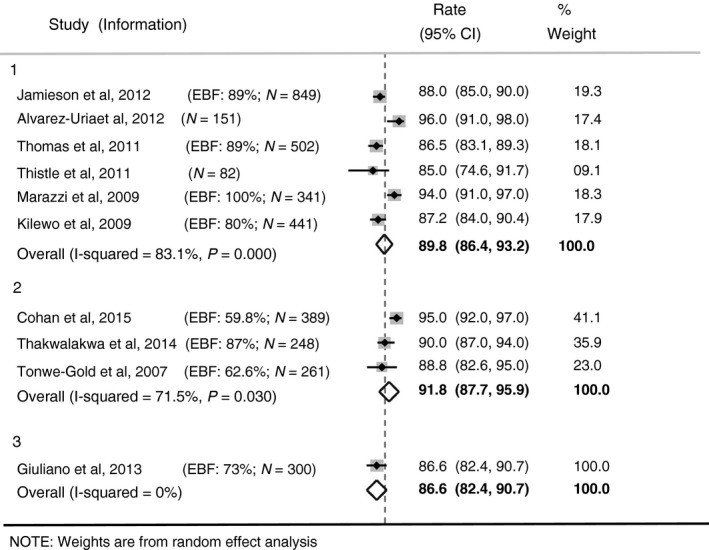
Twelve‐month HIV‐free Survival. Group 1: Mothers on ART up to 6 months postnatally. Group 2: Mothers on Lifelong ART. Group 3: Mixture of Lifelong ART and ART up to 6 months postnatally.

Estimates of 12‐month HIV‐free survival, with confidence intervals, for breastfed infants by duration of maternal ART were obtained from 10 studies (Figure 2). In six studies (Group 1 in Figure 2), HIV‐free survival was reported for infants whose mothers were on ART up to 6 months post‐natally only, with 12‐month estimates ranging from 85% (95% CI 74.6–91.7%) 18 to 96% (95% CI 91–98%) 17. The pooled estimated 12‐month HIV‐free survival was 89.8% (95% CI 86.4–93.2%), with considerable heterogeneity (*I* ^2^ = 83.1%).

In three studies, 12‐month HIV‐free survival was estimated amongst infants whose mothers were on lifelong ART (Group 2 in Figure 2) with estimated HIV‐free survival ranging from 88.8% (95% CI 82.6–95%) 25 to 95% (95% CI 92–97%) 28. The pooled estimate of 12‐month HIV‐free survival was 91.8% (95% CI 87.7–95.9%); heterogeneity was considerable ((*I* ^2^ = 71.5%).

The study by Giuliano 22 (Group 3 in Figure 2), with an estimate of 86.6% (95% CI 82.4–90.7%) included some mothers on ART up to 6 months post‐natally only and others on lifelong ART, but separate estimates were not provided.

#### At age 18 months

**Figure 3:**
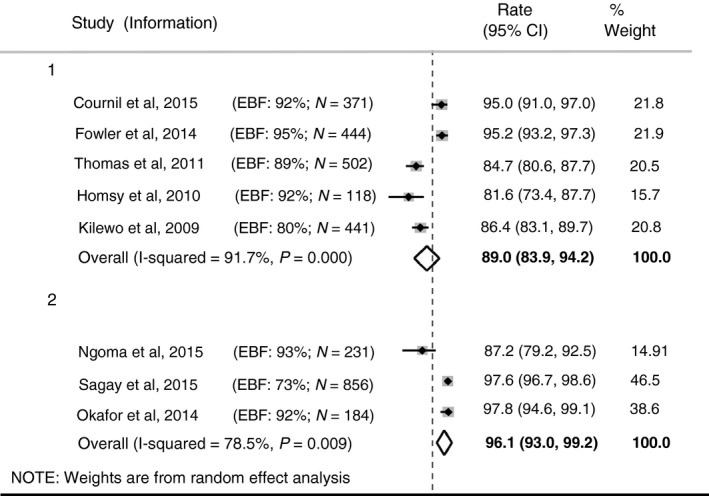
Eighteen‐month HIV‐free survival in children whose mothers breastfed and were on ART. Group 1: Mothers on ART up to 6 months postnatally only. Group 2: Mothers on lifelong ART.

Eight studies provided estimates for 18‐month HIV‐free survival (Figure 3), by duration of maternal ART. In the five studies reporting HIV‐free survival at this age for infants of women who were on ART up to 6 months post‐natally only (Group 1 in Figure 3), HIV‐free survival ranged from 81.6% (95% CI 73.4–87.7%)^19^ to 95.2% (95% CI 93.2–97.3%) 10. The pooled estimate of 18‐month HIV‐free survival for this group was 89.0% (95% CI 83.9–94.2%), with considerable heterogeneity (*I* ^2^ = 91.7%).

In studies where women were on lifelong ART (Group 2 in Figure 3), 18‐month HIV‐free survival estimates ranged from 87.2% (95% CI 79.2–92.5%) 27 to 97.6% (95% CI 96.7–98.6%) 26. The pooled estimated of 18‐month HIV‐free survival for this group was 96.1% (95% CI 93.0–99.2%), again with considerable heterogeneity (*I* ^2^ = 78.5%).

#### At age 24 months

**Figure 4:**
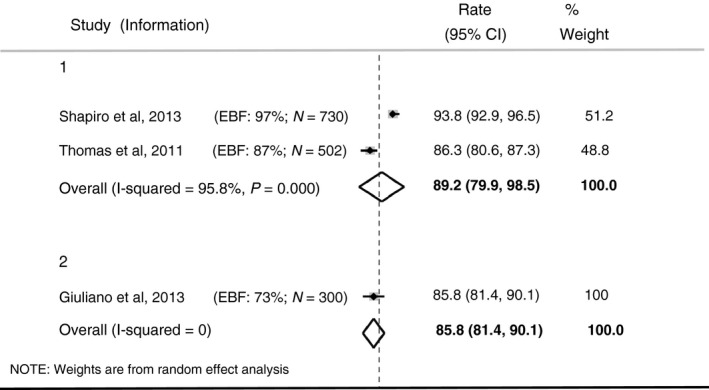
Twenty‐four‐month HIV‐free survival in children whose mothers breastfed and were on ART. Group 1: Mothers on ART up to 6 months postnatally. Group 2: Mixture of mothers, some on lifelong ART, others on ART to 6 months postnatally.

Twenty‐four‐month HV‐free survival estimates from three studies are shown in Figure 4. The estimates in the two studies that reported HIV‐free survival amongst children whose mothers were on ART up to 6 months post‐natally only (Group 1 in Figure 4) were 84.3% (95% CI 80.6–87.3%) 29 and 93.8.0% (95% CI 92.9–96.5%) 31. The pooled estimate for HIV‐free survival at 24 months was 89.2% (95% CI 79.9–98.5%); heterogeneity was considerable (*I* ^2^ = 95.8%).

In the study by Giuliano *et al*. 22, (Group 2 in Figure 4) estimated HIV‐free survival at 24 months was 85.8% (95% CI 81.4% 90.1%), based on a mixed group of infants with respect to mother's ART, some were on ART up to 6 months post‐natally only, while others were on ART for life.

#### At 12, 18 and 24 months

Three studies provided estimates of HIV‐free survival at 12, 18 and 24 months allowing a comparison of HIV‐free survival between 12 and 24 months 22, 23, 29. In all three, HIV‐free survival at 18 months and/or 24 months was not statistically significantly different from the estimate at 12 months. HIV‐free survival estimates were 86.6% (95% CI 82.4%, 90.7%) and 85.8% (95% CI 81.4, 90.1%) at 12 and 24 months, respectively, in Giuliano *et al*. 22, 86.5% (95% CI 83.1%, 89.3%), 84.7% (95% CI 80.6%, 87.7%) and 84.3% (95% CI 80.6%, 87.3%) at 12, 18 and 24 months, respectively, in Thomas *et al*. 29, and in Kilewo *et al*. 23, the estimates were 87.2% (95% CI 84%, 90.4%) at 12 months and 86.4% (95% CI 83.1%, 89.7%) at 18 months.

#### HIV‐free survival by infant feeding modality

**Figure 5:**
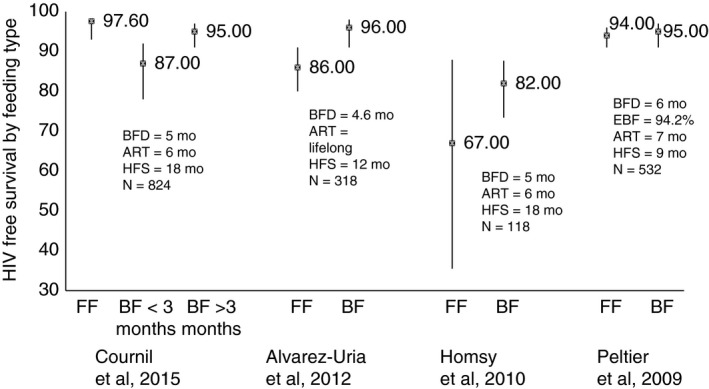
HIV‐free survival by feeding modality in four studies. FF, Formula fed, BF, Breastfed. Cournil *et al.,* 2015 excluded mothers in clinical stage 4 or with CD4 <200 cells/mm^3^. HIV‐free survival excluded endpoints during the first 2 weeks, and was measured at 18 months. Alvarez Uria *et al.,* 2012: HIV free survival was obtained after 8 weeks, and measured at 12 months. Homsy *et al.,* 2010: Mothers received ART when CD4 cell counts ≤250 cells/μl or when with WHO stage III or IV disease. There was no transmission in the study, but four children who died had not been tested. HIV‐free survival was not provided according to feeding modality, but numbers of deaths by feeding were provided. Among 118 children, only nine were formula fed, of whom three died; two received mixed feeding, and both died. Peltier *et al.,* 2009: Mothers with CD4 cell counts ≤350 cells/mm^3^ remained on ART. HIV free survival was measured at 9 months and endpoints were considered from 24 h.

In the Kesho Bora study 8, 9, estimated 18‐month HIV‐free survival was statistically significantly higher amongst formula‐fed (97.6%; 95% CI 93%, 98%) than infants breastfed for less than 3 months (87%; 95% CI 78%, 92%) (Figure 5). The difference between formula‐fed infants (97.6%; 95% CI 93%, 98%) and those breastfed for more than 3 months (95%; 95% CI 91%, 97%) was not statistically significant. In the studies by Alvarez‐Uria 17, reporting estimates at 18 months, Homsy *et al*. 19 at 12 months and Peltier *et al*. 20 at 9 months differences in HIV‐free survival between formula‐fed and breastfed infants were not statistically significant (Figure 5).

#### HIV Transmission

Eight studies provided estimates of death and transmission overall and after 6 months of age, when the infant would have been weaned and mothers were no longer receiving ART. In Ngoma *et al*. 27, there were three peripartum infections, none between 6 weeks and 6 months, and 6 after 6 months. In the studies of both Giuliano *et al*. 22 and Kilewo *et al*. 23 *,* there were eight infections overall, four before weaning at 6 months and four after weaning. In the study by Giuliano *et al*., two of the four mothers who transmitted HIV after weaning were still receiving ART. In the other five studies, there were fewer infections after 6 months of age than before 8, 24, 26, 29, 32, but numbers were again very limited.

#### Mortality

The pattern of infant deaths before and after weaning varied across studies. Jamieson *et al*. 32 reported the same number of deaths before and after weaning (9 of 18 deaths before weaning); Tonwe‐Gold 25 reported one death after and nine deaths before weaning and Ngoma *et al*. 27 reported eight of 20 deaths after weaning. Thomas *et al*. 29, Shapiro *et al*. 31, Kilewo *et al*. [23.] and Marazzi *et al*. [24.] reported higher numbers of deaths after (31 of 49, 22 of 37, 21 of 31 and 7 of 11, respectively) than before weaning. In Shapiro 31, the death rate within 3 months of weaning was significantly higher than during breastfeeding (RR 3.7; 95% CI: 1.3–12.0; *P* = 0.007), and the same was shown comparing those who were weaned before 3 months of age and those aged 3 months or more at weaning (RR = 7.5; 95% CI: 3.2–18.4; *P* < 0.001). Cournil *et al*. 8 did not report any deaths after 6 months, but the risk of dying by 6 months of age in children who stopped breastfeeding before 3 months of age was higher than in those who stopped after more than 3 months (HR 3.94; 95% CI: 1.27–12.27).

### GRADE profile

An evaluation of the overall quality of the combined studies for each outcome is provided in Table S5 using the GRADE approach 5. Study limitations were based on the assessment from the Newcastle–Ottawa Scale and combined with an evaluation of inconsistency, indirectness, imprecision and publication bias. All studies were observational and initially scored as low quality; they were further downgraded for indirectness because their research areas were not directly in line with the research question. Where a pooled analysis was undertaken and a pooled estimate provided, studies were further downgraded for inconsistency when heterogeneity could not be explained. In all groups of studies, there was at least one study with a risk of bias pertaining to lack of detailed information on feeding leading to further downgrading. In one study, the criteria for assessing HIV‐free survival were not clear 21 and this contributed to the downgrading of the quality of the studies grouped together with this study. One study 22 was not downgraded for risk of bias and inconsistency, as it was the single study contributing data in that group.

## Discussion

This systematic review confirms substantially reduced rates of overall and post‐natal HIV transmission and mortality up to 24 months of age when pregnant and breastfeeding women receive ART. The difference in HIV‐free survival between infants of mothers receiving ART for PMTCT only or for life diverged beyond 6 months post‐delivery and became particularly evident at 18 months. At this time‐point, the estimated HIV‐free survival was about 89% when mothers ceased ART at 6 months, and 96% when mothers continued ART for life. The risk of transmission through breastfeeding continued beyond the recommended 6 months and after cessation of maternal ART 1.

Our results thus confirm the importance of breastfeeding for infant survival and the effectiveness of ART in reducing the risk of mother‐to‐child HIV transmission. In most studies (5 of 8), there were more infant deaths after weaning than before, but in only one of eight studies was the number of HIV transmissions after 6 months higher than before, which together would suggest that HIV‐free survival in the first year of life is substantially affected by increased mortality following early cessation of breastfeeding rather than by increased infections that may occur in infants of women who had stopped taking ART 8, 22, 23, 24, 25, 27, 29, 31, 32.

Our review, although systematic in nature, had limitations. There was considerable statistical heterogeneity in all pooled estimates, and the overall assessment of quality of the evidence was very low. The low quality of evidence was mainly attributed to the fact that all studies included in the analysis were observational and the evidence was further downgraded due to indirectness, and lack of data on feeding history and adherence to ART. The absolute estimates of HIV‐free survival should thus be interpreted with caution and consider both the mean value and the confidence interval limits. Further, we were unable to formally allow for factors known to be associated with transmission and/or child survival, and heterogeneity between studies could be partially due to differences in other factors associated with child mortality. There was limited information on children born to women on lifelong ART where ART was initiated before advanced HIV progression, and most of the women on lifelong ART in the studies that reported such information would have been eligible as per the national and WHO guidelines at the time of the study 16, 21, 25, 26, 27, 28.

Increased HIV‐free survival in the studied populations was linked to early initiation of maternal ART in pregnancy, continued post‐partum and breastfeeding. Increased infant/child mortality after 6 months of life may be attributed to early cessation of breastfeeding and could possibly contribute to reduced HIV‐free survival in non‐breastfed HIV‐exposed infants. Recent findings from a randomised trial, evaluating the use of two different infant antiviral drugs in the prevention of post‐natal HIV acquisition, showed HIV‐free survival similar to what we present here with maternal ART 33; infant ARV may therefore be an alternative intervention to achieve the prevention of mother‐to‐child transmission in cases where maternal ART is not possible or not being adhered to. Our findings suggest prolonged breastfeeding for women on ART for life is beneficial in terms of HIV‐free survival at least up to 2 years of age. With expanding eligibility criteria, and WHO now recommending initiation of ART for life immediately after HIV diagnosis, most women will be on ART for life from before or early pregnancy.

## Supporting information


**Table S1** Modified Newcastle – Ottawa Quality Assessment Scale

**Table S2** Excluded papers (Studies are based on published papers, which were screened based on the search criteria in Appendix 1. Some studies are additional outputs of larger studies that produced further papers and reports not considered in this study.) with reason for exclusion, after full text screening

**Table S3** Included Studies: Descriptive information of studies providing information on breastfeeding and ART

**Table S4** Assessment of studies HIV‐free Survival in breastfed infants whose mothers were on ART based on the Modified Newcastle‐Ottawa Scale

**Table S5** Grade Evidence Profiles
