# Supplementary material for: HIV‐free survival at 12–24 months in breastfed infants of HIV‐infected women on antiretroviral treatment
Source: Trop Med Int Health. 2016 May 24;21(7):820–8. doi: 10.1111/tmi.12710 (PMC5096069; doi:10.1111/tmi.12710)
Supplement: Supplementary file 1 — Table S1 Modified Newcastle – Ottawa Quality Assessment Scale [file TMI-21-820-s001.docx]

**Supplementary Table 1** Modified NEWCASTLE - OTTAWA QUALITY ASSESSMENT SCALE

Note: A study can be awarded a maximum of one star for each numbered item within the Selection and Outcome categories. The item Comparability of cohorts assesses whether exposed and non-exposed individuals are matched in the design and risk for the exposure of interest is adjusted for confounders. In this study all mothers are exposed to ART, and the outcome is HIV free-survival (not relative risk or odds ratio), which is not controlled for confounders or covariates, therefore item comparability was not applied in the quality assessment.

**Selection**

1) Representativeness of the exposed cohort

Assesses whether the women on ART in the study are representative of women on ART in general

a) truly representative of the average women on ART in the community ****

b) somewhat representative of the average woman on ART in the community ****

c) selected group of users

d) no description of the derivation of the cohort

2) Ascertainment of exposure (ART)

a) secure record (e.g. clinical records) ****

b) structured interview ****

c) written self report

d) no description

3) Adherence to ART

a) Adherence reported in sufficient detail and adherence rates at end of study are high ****

b) Adherence reported in sufficient detail, non-adherence <20% and unlikely to introduce bias****

c) Not described

4) Treatment eligibility

a) lifelong ART for all women irrespective of HIV disease progression ****

b) ART provided for PMTCT and only for 6 months or longer if breastfeeding continues

c) Eligibility of ART on the basis of CD4 count or disease progression

5) Ascertainment of exposure (BF)

a) secure record (eg clinical records, close follow-up) ****

b) structured interview ****

c) written self report

d) no description

6) Duration of BF

a) Clear report of Exclusive breastfeeding up to 6 months and continue BF for 1 year ****

b) Breastfeeding cessation at maximum 6 months

c) not described in detail

**Outcome**

1) Assessment of outcome

Outcome is infant death or transmission combined into HIV-free survival. This item assesses whether the information regarding infection was assessed per protocol visit and laboratory procedures, and child survival per clinical records. Where studies only report HIV transmission or only death and not HIV-free survival estimates, they do not score on this item.

a) independent assessment ****

b) record linkage with HIV clinical programmes ****

c) self-report

d) no description

2) Timing of outcome assessment

Infection/survival is normally assessed various times during a trial, and information should be provided on the cohorts nested in an HIV programme. Where HIV-free survival estimates are provided at more than one age point a study scores one star on this item. Studies, which only report numbers of infections occurring between age points without providing a denominator at risk, do not score on this item.

a) At least at two different points ****

b) Only at one time-point

3) Outcome stratified by feeding type

a) Yes ****

b) No

4) Was follow-up long enough for outcomes to occur

a) yes (select an adequate follow up period for outcome of interest) ****

b) no

5) Adequacy of follow up of cohorts

a) complete follow up - all subjects accounted for ****

b) subjects lost to follow up unlikely to introduce bias - small number lost - <20%****

c) follow up rate < 20% and no description of those lost

d) no statement
