# Supplementary material for: HIV‐free survival at 12–24 months in breastfed infants of HIV‐infected women on antiretroviral treatment
Source: Trop Med Int Health. 2016 May 24;21(7):820–8. doi: 10.1111/tmi.12710 (PMC5096069; doi:10.1111/tmi.12710)
Supplement: Supplementary file 2 — Table S2 Excluded papers (Studies are based on published papers, which were screened based on the search criteria in Appendix 1. Some studies are additional outputs of larger studies that produced further papers and reports not considered in this study.) with reason for exclusion, after full text screening [file TMI-21-820-s002.docx]

Supplementary Table 2 Excluded papers ^^[[1]](#footnote-1)^^with reason for exclusion, after full text screening

| No. | Reference | Reason for Exclusion |
| --- | --- | --- |
| 1. | Anoje C, Aiyenigba B, Suzuki C, Badru T, Akpoigbe K, Odo M, et al. Reducing mother-to-child transmission of HIV: findings from an early infant diagnosis program in south-south region of Nigeria. BMC Public Health. 2012;12:184. | The study combines both groups of women on different types of ARV, and do not provide infant HIV free survival when mothers are breastfeeding and on ART. |
| 2. | Becquet R, Bequet L, Ekouevi DK, Viho I, Sakarovitch C, Fassinou P, et al. Two-year morbidity-mortality and alternatives to prolonged breast-feeding among children born to HIV-infected mothers in Cote d'Ivoire. PLoS Med. 2007;4(1):e17 | Mothers were not on ART, but on dual ARV with single dose NVP on labour. |
| 3. | Becquet R, Ekouevi DK, Menan H, Amani-Bosse C, Bequet L, Viho I, et al. Early mixed feeding and breastfeeding beyond 6 months increase the risk of postnatal HIV transmission: ANRS 1201/1202 Ditrame Plus, Abidjan, Cote d'Ivoire. Preventive Medicine. 2008;47(1):27-33. | Mothers were not on ART, but on dual ARV with single dose NVP on labour. |
| 4. | Binagwaho A, Pegurri E, Drobac PC, Mugwaneza P, Stulac SN, Wagner CM, et al. Prevention of mother-to-child transmission of HIV: cost-effectiveness of antiretroviral regimens and feeding options in Rwanda. PLoS One. 2013;8(2):e54180 | Mothers are on short course ART, and does not provide HIV free survival. Focused on costs. |
| 5. | Chi BH, Musonda P, Lembalemba MK, Chintu NT, Gartland MG, Mulenga SN, et al. Universal combination antiretroviral regimens to prevent mother-to-child transmission of HIV in rural Zambia: a two-round cross-sectional study. Bulletin of the World Health Organization. 2014;92(8):582-92. | Provides only total HIV free survival, not total number of mothers on ART or infection only by ART. |
| 8. | Derebe G, Biadgilign S, Trivelli M, Hundessa G, Robi ZD, Gebre-Mariam M, et al. Determinant and outcome of early diagnosis of HIV infection among HIV-exposed infants in southwest Ethiopia. BMC research notes. 2014;7:309 | No rates for breastfeeding and ART together were provided. |
| 10. | Goga AE, Dinh TH, Jackson DJ, Lombard C, Delaney KP. First population-level effectiveness evaluation of a national programme to prevent HIV transmission from mother to child, South Africa. 2015;69(3):240-8 | It is very early diagnosis (4-6 weeks). |
| 11. | Gray GE, Urban M, Chersich MF, Bolton C, van Niekerk R, Violari A, et al. A randomized trial of two postexposure prophylaxis regimens to reduce mother-to-child HIV-1 transmission in infants of untreated mothers. AIDS (London, England). 2005;19(12):1289-97 | Mothers were not on ART, but on dual ARV. |
| 12. | Kagaayi J, Gray RH, Brahmbhatt H, Kigozi G, Nalugoda F, Wabwire-Mangen F, et al. Survival of infants born to HIV-positive mothers, by feeding modality, in Rakai, Uganda. PLoS One. 2008;3(12):e387 | Mothers on different type of antiretroviral therapy. Not possible to identify HIV transmission and death by mothers on ART. |
| 14. | Kouanda S, Tougri H, Cisse M, Simpore J, Pietra V, Doulougou B, et al. Impact of maternal HAART on the prevention of mother-to-child transmission of HIV: results of an 18-month follow-up study in Ouagadougou, Burkina Faso. AIDS care. 2010;22(7):843-50 | Only 8 mothers on ART were breastfeeding on the first exam. |
| 15. | Kuhn L, Aldrovandi GM, Sinkala M, Kankasa C, Semrau K, Kasonde P, et al. Differential effects of early weaning for HIV-free survival of children born to HIV-infected mothers by severity of maternal disease. PLoS One. 2009;4(6):e6059 | Mothers received single-dose nevirapine. |
| 16 | Leroy V, Ekouevi DK, Becquet R, Viho I, Dequae-Merchadou L, Tonwe-Gold B, et al. 18-month effectiveness of short-course antiretroviral regimens combined with alternatives to breastfeeding to prevent HIV mother-to-child transmission. PLoS One. 2008;3(2):e1645 | Mothers were not on ART, but on dual ARV with single dose NVP on labour. |
| 17. | Magoni M, Bassani L, Okong P, Kituuka P, Germinario EP, Giuliano M, et al. Mode of infant feeding and HIV infection in children in a program for prevention of mother-to-child transmission in Uganda. AIDS (London, England). 2005;19(4):433-7 | Mother receiving short course ART. |
| 18. | Mandala J, Moyo T, Torpey K, Weaver M, Suzuki C, Dirks R, et al. Use of service data to inform pediatric HIV-free survival following prevention of mother-to-child transmission programs in rural Malawi. Bmc Public Health. 2012;12 | Mothers on single dose NVP. |
| 19. | Minniear TD, Girde S, Angira F, Mills LA, Zeh C, Peters PJ, et al. Outcomes in a cohort of women who discontinued maternal triple-antiretroviral regimens initially used to prevent mother-to-child transmission during pregnancy and breastfeeding--Kenya, 2003-2009. PLoS One. 2014;9(4):e93556 | Mothers discontinued ART after labour. |
| 20. | Mwendo EM, Mtuy TB, Renju J, Rutherford GW, Nondi J, Sichalwe AW, et al. Effectiveness of prevention of mother-to-child HIV transmission programmes in Kilimanjaro region, northern Tanzania. Tropical medicine & international health : TM & IH. 2014;19(3):267-74 | Almost 50% of losses on the first PCR test, and only 7 children completed 18 months follow-up. |
| 21. | Nagot N, Kankasa C, Meda N, Hofmeyr J, Nikodem C, Tumwine JK, et al. Lopinavir/Ritonavir versus Lamivudine peri-exposure prophylaxis to prevent HIV-1 transmission by breastfeeding: the PROMISE-PEP trial Protocol ANRS 12174. BMC infectious diseases. 2012;12:246 | Mothers included on the study were not eligible for ART. |
| 22. | Nlend AEN, Ekani BB. Preliminary assessment of breastfeeding practices in HIV 1-infected mothers (prior to weaning) under the Djoungolo programme on the prevention of mother-to-child transmission of HIV. Journal of tropical pediatrics. 2010;56(6):436-9 | The paper focus on breastfeeding, mastitis and transmission, and assessment of transmission done at 13 weeks. |
| 23. | Nyandiko WM, Otieno-Nyunya B, Musick B, Bucher-Yiannoutsos S, Akhaabi P, Lane K, et al. Outcomes of HIV-exposed children in western Kenya: efficacy of prevention of mother to child transmission in a resource-constrained setting. Journal of acquired immune deficiency syndromes (1999). 2010;54(1):42-50 | Not possible to extract transmission and death for ART and BF together. |
| 24. | Omer SB. Twelve-month follow-up of Six Week Extended Dose Nevirapine randomized controlled trials: differential impact of extended-dose nevirapine on mother-to-child transmission and infant death by maternal CD4 cell count. AIDS (London, England). 2011;25(6):767-76 | Most mothers were not on ART. |
| 25. | Palombi L, Marazzi MC, Voetberg A, Magid NA. Treatment acceleration program and the experience of the DREAM program in prevention of mother-to-child transmission of HIV. AIDS (London, England). 2007;21 Suppl 4:S65-71 | Same data from DREAM study as another selected study. |
| 26. | Read JS. Prevention of mother-to-child transmission of HIV: antiretroviral strategies. Clinics in perinatology. 2010;37(4):765-76, viii | Very small number of mothers on ART. |
| 27. | Seth A, Chandra J, Gupta R, Kumar P, Aggarwal V, Dutta A. Outcome of HIV exposed infants: experience of a regional pediatric center for HIV in North India. Indian J Pediatr. 2012;79(2):188-93 | Very small number of mothers on ART, and not provided information if those mothers were breastfeeding or not. |
| 28. | Shah M, Johns B, Abimiku Al, Walker DG. Cost-effectiveness of new WHO recommendations for prevention of mother-to-child transmission of HIV in a resource-limited setting. AIDS (London, England). 2011;25(8):1093-102. | Study based on models of % of adherence in Nigeria. |
| 31. | Simpore J, Pietra V, Pignatelli S, Karou D, Nadembega WM, Ilboudo D, et al. Effective program against mother-to-child transmission of HIV at Saint Camille Medical Centre in Burkina Faso. Journal of medical virology. 2007;79(7):873-9 | Very small number of breastfed children. |
| 32. | Stringer JS, Stinson K, Tih PM, Giganti MJ, Ekouevi DK, Creek TL, et al. Measuring coverage in MNCH: population HIV-free survival among children under two years of age in four African countries. PLoS Med. 2013;10(5):e1001424 | Only provide HIV free survival for mothers on ART or dual ARV together. |
| 33. | Taha TE, Li Q, Hoover DR, Mipando L, Nkanaunena K, Thigpen MC, et al. Postexposure Prophylaxis of Breastfeeding HIV-Exposed Infants With Antiretroviral Drugs to Age 14 Weeks: Updated Efficacy Results of the PEPI-Malawi Trial. Jaids-Journal of Acquired Immune Deficiency Syndromes. 2011;57(4):319-25 | Mothers not on ART, comparison among dual therapy |
| 34. | Torpey K, Kabaso M, Weaver MA, Kasonde P, Mukonka V, Bweupe M, et al. Infant feeding options, other nonchemoprophylactic factors, and mother-to-child transmission of HIV in Zambia. Journal of the International Association of Physicians in AIDS Care (Chicago, Ill : 2002). 2012;11(1):26-33 | No data for mother on ART and breastfeeding together |
| 35. | Torpey K, Kasonde P, Kabaso M, Weaver MA, Bryan G, Mukonka V, et al. Reducing pediatric HIV infection: estimating mother-to-child transmission rates in a program setting in Zambia. Journal of acquired immune deficiency syndromes (1999). 2010;54(4):415-22 | No data for mother on ART and breastfeeding together |
| 36. | van Lettow M, Bedell R, Landes M, Gawa L, Gatto S, Mayuni I, et al. Uptake and outcomes of a prevention-of mother-to-child transmission (PMTCT) program in Zomba district, Malawi. BMC Public Health. 2011;11:426 | Very small number of mothers on ART |

1. Studies are based on published papers, which were screened based on the search criteria in Appendix 1. Some studies are additional outputs of larger studies that produced further papers and reports not considered in this study. [↑](#footnote-ref-1)
