# Supplementary material for: HIV‐free survival at 12–24 months in breastfed infants of HIV‐infected women on antiretroviral treatment
Source: Trop Med Int Health. 2016 May 24;21(7):820–8. doi: 10.1111/tmi.12710 (PMC5096069; doi:10.1111/tmi.12710)
Supplement: Supplementary file 3 — Table S3 Included Studies: Descriptive information of studies providing information on breastfeeding and ART [file TMI-21-820-s003.docx]

Supplementary Table 3 Included Studies: Descriptive information of studies providing information on breastfeeding and ART

| **Cohorts embedded on RCTs** | | | | | | | | | | |
| --- | --- | --- | --- | --- | --- | --- | --- | --- | --- | --- |
| **First Author/Study** | **Place of study** | **Randomised for** | | **Feeding** | **Beginning/ end ART** | **Breast-**  **feeding duration** | **Time evaluation** | **N** | **Extracted information** | |
|  |  | **ART** | **Other** |  |  |  |  |  | **HFS** | **Included** |
| Kesho Bora Study (Cournil, 2015-2013; de Vicenzi, 2011-2010 | Burkina Faso, Kenya and South Africa | HAARTscARV† |  | BF<3 mo, BF≥ 3mo, RF | 34wk/ cessation BF | 6 mo | 12, 18 mo | 371 | Given | Mortality and Transmission from 2 weeks |
| HPTN046 trial (Fowler, 2014; Coovadia, 2012) | South Africa, Tanzania, Uganda and Zimbabwe | HAART no ARV | Infant NVP or not | All BF | From first antenatal visit/ 6 mo | 6 mo | 18 mo | 1527 | Given | Transmission from 6 weeks |
| Mma Bana Study (Shapiro et al, 2013,2010 and 2009) | Botswana | 2 types of HAART† |  | Majority BF | 26 or 34 wks/ 6 mo‡ | 6 mo | 24 mo | 730 | Given | All transmission and mortality |
| Jamieson, 2012 | Antenatal clinics in Malawi | HAART† or infant NVP | Nutritional intervention | Majority BF | 30wks or less/ 6 mo | 6 mo | 12 mo | 849 | Given | All transmission and mortality |
| Kisumu Breastfeeding Study (Okanda, 2014; Thomas, 2011) | Kenya (antenatal clinics) | HAART |  | All BF | 34 wks/ 6 mo‡ | 6 months | 12, 18, 24 mo | 502 | Given | All transmission and mortality |
| Cohan^b^, 2015 | Uganda, antental clinics in Tororo District | 2 types of HAART |  | Majority BF | 30 wk or less/life long | 1 year | 12 mo | 389 | Given | All transmission and mortality |
| Thakwalakwa^a^, 2014 | Malawi (Thyolo District Hospital) | Only HAART | Nutritional intervention | All BF | From first antenatal visit/ lifelong | 6 mo | 12 mo | 248 | Given | Mortality and Transmission from 6 weeks |
| **Observational studies** | | | | | | | | | | |
| Okafor, 2014 | Nigeria (Enugu State University Teaching Hospital Parklane) | HAART |  | BF, MF, RF | 14 wk/life long | 12 mo | 18 mo | 184 | Calculated | Not clear |
| DREAM study (Giuliano, 2013; Palombi, 2007) | Malawi (two ante natal clinics) | HAART* |  | All BF | 1st tremester and lifelong (CD4+<350) or week 25/ 6 mo or end BF | 4.5 mo | 12 and 24 mo | 300 | Given | All transmission and excluded death in 24h (n=3) |
| Alvarez-Uria^a^, 2012 | India (3 hospitals in Antapur) | HAART |  | BF and RF | From first antenatal visit / 6 mo (BF), post labour (NBF) | 6 mo | 12 mo | 318 | Given | All transmission and mortality from the 1^st^ week |
| Thistle^a^, 2015 | Zimbabwean (Salvation Army Hospital) | HAART |  | All BF | Between 14 and 36 wks/ 6 mo | 6 mo | 12 mo | 82 | Calculated | All transmission and mortality |
| Homsy^a^, 2010 | Uganda (Tororo and Busia Districts) | HAART* |  | All BF | From first antenatal visit/ 6 mo | 3-6 mo | 18 mo | 118 | Calculated | All transmission and mortality |
| Peltier^a^, 2009 | Rwanda (four government-run health facilities) | HAART |  | BF and RF | 28 wks / 7 mo‡ | 6 mo | 9 mo | 532 | Given | All transmission and mortality from 48h |
| Marazzi, 2009 | Mozambique | HAART |  | All BF | 15 wks/ 6 mo‡ | 5 mo | 12 mo | 341 | Given | All transmission and mortality |
| Kilewo, 2009 | Tanzania (Dar es Salam) | HAART |  | All BF | 34 wks/ 6 mo | 6 mo | 9, 12, 18 mo | 441 | Given | All transmission and mortality |
| Tonwe-Gold, 2007 | Cote d’Ivoire (2 community-based antenatal clinics in Abidjan) | HAART and scARV or sdNVP* |  | BF and RF | 24 wk/ lifelong | 6 mo | 12, 18 mo | 261 | Given | All transmission and mortality |
| Sagay, 2015 | Nigeria | All HAART |  | All BF | Lifelong | 1 year | 18 mo | 856 | Given | All transmission and mortality |
| Ngoma, 2015 | Zambia | All HAART |  | All BF | 14 wks-lifelong | 1 year | 12 mo | 231 | Given | All transmission and mortality |

^a^ Studies performed in rural environment

^b^ The study was designed to test the hypothesis that lopinavir/ritonavir would reduce placental malaria

†Mothers on clinical stage 4 or CD4 <200 cells/mm^3^ were excluded

‡Mothers with CD4 count of <200 cells/mm3 or stage III or IV remained on HAART throughout the study, and those who subsequently met the criteria after stopping ARVs were restarted, or when CD4 cell counts ≤350 cells/mm^3^ (Peltier, Marazzi)

*Mothers on HAART based on disease progression or low CD4+ count
