# Supplementary material for: HIV‐free survival at 12–24 months in breastfed infants of HIV‐infected women on antiretroviral treatment
Source: Trop Med Int Health. 2016 May 24;21(7):820–8. doi: 10.1111/tmi.12710 (PMC5096069; doi:10.1111/tmi.12710)
Supplement: Supplementary file 4 — Table S4 Assessment of studies HIV‐free Survival in breastfed infants whose mothers were on ART based on the Modified Newcastle‐Ottawa Scale [file TMI-21-820-s004.docx]

Supplementary Table 4. Assessment of studies HIV-free Survival in breastfed infants whose mothers were on ART based on the Modified Newcastle-Ottawa Scale

| **Author** | **Country** | **Selection** | **Outcome** |
| --- | --- | --- | --- |
| Ngoma et al, 2015([27](#_ENREF_27)) | Zambia | ****** | ** |
| Sagay et al, 2015([26](#_ENREF_26)) | Nigeria | **** | ** |
| Cournil et al, 2015([8](#_ENREF_8)) | Burkina Faso, Kenia and South Africa | ** | *** |
| Cohan et al, 2015([28](#_ENREF_28)) | Uganda | **** | ** |
| Thakwalakwa et al, 2014 ([16](#_ENREF_16)) | Malawi | ** | ** |
| Okafor et al, 2014([21](#_ENREF_21)) | Nigeria | *** | * |
| Giuliano et al, 2013 ([22](#_ENREF_22)) | Malawi | ** | *** |
| Shapiro et al, 2013([31](#_ENREF_31))] | Botswana | *** | ** |
| Coovadia et al, 2012([30](#_ENREF_30)) | South Africa, Tanzania, Uganda and Zimbabwe | ** | * |
| Jamieson et al, 2012([32](#_ENREF_32)) | Malawi | **** | ** |
| Alvarez-Uria et al, 2012([17](#_ENREF_17)) | India | ** | **** |
| Thomas et al, 2011([29](#_ENREF_29)) | Kenya | **** | *** |
| Thistle et al, 2011([18](#_ENREF_18)) | Zimbabwe | ** | ** |
| Homsy, 2010([19](#_ENREF_19)) | Uganda | *** | ** |
| Peltier, 2009([20](#_ENREF_20)) | Rwanda | **** | *** |
| Marazzi, 2009 ([24](#_ENREF_24)) | Mozambique | ** | *** |
| Kilewo, 2009([23](#_ENREF_23)) | Tanzania | *** | *** |
| Tonwe-Gold, 2007([25](#_ENREF_25)) | Cote d'Ivoire | *** | **** |
