# Supplementary material for: HIV‐free survival at 12–24 months in breastfed infants of HIV‐infected women on antiretroviral treatment
Source: Trop Med Int Health. 2016 May 24;21(7):820–8. doi: 10.1111/tmi.12710 (PMC5096069; doi:10.1111/tmi.12710)
Supplement: Supplementary file 5 — Table S5 Grade Evidence Profiles [file TMI-21-820-s005.docx]

**Supplementary Table 5** Grade Evidence Profiles

**Question**: HIV-free survival in Breastfed Infants of mothers on ART ^1^

**Setting**: India, Botswana, Burkina Faso, Ivory Coast, Kenya, Malawi, Mozambique, Nigeria, Rwanda, South Africa, Tanzania, Uganda, Zambia and Zimbabwe

| **Quality assessment** | | | | | | | **№ of patients** | **Effect** | **Quality** | **Importance** |
| --- | --- | --- | --- | --- | --- | --- | --- | --- | --- | --- |
| **№ of studies** | **Study design** | **Risk of bias** | **Inconsistency** | **Indirectness** | **Imprecision** | **Other considerations** | **Total number of breastfed infants of mothers on ART** | **Percentage of HIV Free Survival (95% CI)** |  |  |
| HIV Free Survival at 12 Months: Mothers on ART up 6 months postnatal | | | | | | | | | | |
| 6 | observational studies ^2^ | serious ^3^ | serious ^4^ | serious ^5^ | not serious | none | 2533 | 89.8% (86.9%, 93.2%) | ⨁◯◯◯ VERY LOW ^4 5 6^ | CRITICAL |
| HIV Free Survival at 12 Months: Mothers on lifelong ART | | | | | | | | | | |
| 3 | observational studies ^6^ | serious ^7^ | serious ^8^ | serious ^9^ | not serious | none | 1198 | 91.9 %( 87.7%, 95.9%) | ⨁◯◯◯ VERY LOW ^8 9 10^ | CRITICAL |
| HIV Free Survival at 12 Months: Mixture of mothers some on lifelong ART and others on ART up to 6 months postnatal | | | | | | | | | | |
| 1 | observational studies ^10^ | not serious | not serious | serious ^11^ | not serious | none | 300 | 86.6% (82.4%, 90.7%) | ⨁◯◯◯ VERY LOW ^12^ | CRITICAL |
| HIV Free Survival at 18 Months: Mothers on ART up to 6 months postnatal | | | | | | | | | | |
| 5 | observational studies ^12^ | serious ^13^ | serious ^14^ | serious ^15^ | not serious | none | 2905 | 89.0% (83.9%, 94.2%) | ⨁◯◯◯ VERY LOW ^14 15 16^ | CRITICAL |
| HIV Free Survival at 18 Months: Mothers on lifelong ART | | | | | | | | | | |
| 3 | observational studies ^16^ | very serious ^17^ | serious ^18^ | serious ^19^ | not serious | none | 1271 | 96.1% (93.0%, 99.2%) | ⨁◯◯◯ VERY LOW ^18 19 20^ | CRITICAL |
| HIV Free Survival at 24 Months: Mothers on ART up 6 months postnatal | | | | | | | | | | |
| 2 | observational studies ^20^ | serious ^21^ | serious ^22^ | serious ^23^ | not serious | none | 1232 | 89.2% (79.9%, 98.5%) | ⨁◯◯◯ VERY LOW ^22 23 24^ | CRITICAL |
| HIV Free Survival at 24 months: Mixture of mothers, some on lifelong ART and others on ART up 6 months postnatal | | | | | | | | | | |
| 1 | observational studies ^10^ | not serious | not serious | serious ^11^ | not serious | none | 300 | 85.8% (81.4%, 90.1%) | ⨁◯◯◯ VERY LOW ^12^ | CRITICAL |

MD – mean difference, RR – relative risk

1. The estimate of HIV-free Survival is a total for all infants in the studies where the majority were breastfed.
2. Four studies; Alvarez-Uria et al 2012, Thistle et al. 2011, Marazzi et al. 2009 and Kilewo et al. 2009 were cohort studies, whilst the studies by Jamieson et al. 2012 and Thomas et al. 2011 were cohorts embedded in randomised control trials.
3. Risk of bias: We downgraded once due to potential selection bias for lack of detailed feeding history in Marazzi et al. 2009, Thistle et al. 2011 and Alvarez-Uria et al. 2012 and 20% loss to follow up in Jamieson et al. 2012 and 18.3% loss to follow up in Thistle et al. 2011.
4. Inconsistency: We downgraded once due substantial heterogeneity in the pooled estimate, I squared= 83%. HIV Free Survival ranged from 85% (95% CI 75%, 92%) Thistle et al. 2011 to 96 %( 95% CI 91% to 98%) Alvarez-Uria et al, 2012.
5. Indirectness: We downgraded once because studies’ research questions were not in line with the PICO question and they covered different types of co-interventions. In Thistle et al 2011, Marazzi et al. 2009 and Kilewo et al. 2009 the only intervention was ART, Jamieson et al. 2012 compared HIV Free Survival in children whose mothers were on ART, infants on NVP and a control group and the three groups were further divided into those who were on a maternal nutrition supplement and those that weren’t. In Alvarez-Uria et al. 2012 all women were on ART but newborns were also given prophylaxis. In Thomas et al. 2011 all mothers were on ART but all infants received a single dose of NVP within 72 hours and TMP/SMX from 6 weeks. Studies also varied with regard to the outcomes of interest of indication, timing of initiation of maternal ART and breastfeeding recommendations and practice. There were no comparative studies.
6. The study by Tonwe-Gold et al. 2007 was a cohort study whilst the studies by Cohan et al. 2015 and Thakwalakwa et al. were cohort studies embedded within randomised control trials.
7. Risk of bias: We downgraded once due to the potential selection bias for lack of detailed feeding history in Tonwe-Gold et al. 2007 and Thakwalakwa et al. 2011.
8. Inconsistency: We downgraded once due to substantial heterogeneity in the pooled estimate, I squared=81.2%. HIV Free Survival estimates ranged from 89% (95% CI 83%, 95%) Tonwe-Gold et al. 2007 to 95 %( 95% CI 92% to 97%) Cohan et al. 2015.
9. Indirectness: We downgraded once, as the studies’ questions were not in line with the PICO question and covered different types of co-interventions. In the study by Tonwe-Gold et al. 2007, all women were on ART and infants received sdNVP at 3 days of life and 1 week of ZDV syrup. In the study by Cohan et al. 2015, mothers were randomised to receive two different types of ART. One group received efavirenz whilst the other received lopinavir/rotonavir and all women received trimethoprim-sulfamethoxazole prophylaxis. In Thakwalakwa et al. all mothers were on ART but at 6 months infants were randomised to receive either milk powder or a ready to use food comprising of peanut paste, skimmed powder, sugar and vegetable oil. Studies also varied with regard to the outcomes of interest of indication, timing of initiation of maternal ART and breastfeeding recommendations and practice. There were no comparative studies
10. The study by Giuliano et al. 2013 was a retrospective cohort study. ART stopped at 6 months for those with CD4+ count greater than 350/mm^3 but continued in those with a CD4+ count less than 350/mm^3
11. Indirectness: We downgraded once since the study research question was not in line with the PICO question. The study assessed HIV Free Survival amongst breastfeeding infants whose mothers were on ART
12. The studies by Cournil et al. 2015, Covaadia et al. 2012, Thomas et al. 2011 were cohort studies embedded in randomised control trials whilst Homsy et al. 2010 and Kilewo et al. 2009, were cohort studies.
13. Risk of bias. We downgraded once due to potential selection bias for lack of feeding history in the study by Thomas et al. 2011.
14. Inconsistency: We downgraded once due to substantial heterogeneity in the pooled estimate, I squared=91.4%. HIV Free Survival ranged from 82 %( 95% CI 73%, 88%) Homsy et al. 2010 to 96 %( 95% CI 94%, 99%).
15. Indirectness: We downgraded once because the studies’ research question was not in line with the PICO question and covered different types of co-interventions; In Cournil et al. 2015 women were randomised to receive triple ARV prophylaxis or short course ARV. In Coovadia et al. 2012, infants were randomised to receive either extended nevirapine prophylaxis or placebo until 6 months or until breastfeeding cessation. In Thomas et al. 2011 all mothers were on ART but all infants received a single dose of NVP within 72 hours and TMP/SMX from 6 weeks. In Homsy et al. all women were on ART and infants received a single dose of NVP within 72 hours of birth. The NVP was later supplemented with AZT syrup during the course of the study. Eligibility for ART in Homsy et al. 2010 was CD4 cell counts equal or les s than 250 cell/uL or WHO Stage 3 or 4. In Kilewo et al. 2009 all mothers were on ART and infants received ZDV+3TC for 1 week after birth. Studies also varied with regard to the outcomes of interest of indication, timing of initiation of maternal ART and breastfeeding recommendations and practice. There were no comparative studies.
16. The studies by Ngoma et al. 2015, Sagay et al. 2015 and Okafor et al. 2014 were cohort studies
17. Risk of Bias: We downgraded twice because Sagay et al. 2015 had a problem with lack of detailed feeding history and Okafor et al. 2014 did not provide a criteria of how they estimated HIV Free survival.
18. Inconsistency: We downgraded once because of substantial heterogeneity in the pooled estimate, I squared= 82%. HIV Free survival ranged from 87 %( 95% CI 79%, 92%) Ngoma et al. 2015 to 98 %( 95% 97%, 99%) Sagay et al. 2015.
19. Indirectness: We downgraded once because the studies’ research questions were not in line with the PICO question and there were slight differences in the co-interventions. In Ngoma et al. 2015 all women were on ART, in Sagay et al. 2015 and Okafor et al. apart from all women being on ART, HIV exposed infants received NVP from birth up to 6 weeks.
20. The two studies by Thomas et al. 2011 and Shapiro et al. 2013 were cohorts embedded within randomised control trials
21. Risk of bias. We downgraded once for potential risk of selection bias due to lack of detailed feeding history in the study by Thomas et al. 2011
22. Inconsistency: We downgraded once due to substantial heterogeneity, I squared=97%. HIV free Survival was 84.35 (95% CI 81%, 87%) in the study by Thomas et al. 2010 and 94 %( 95% CI 92%, 96%) in the study by Shapiro et al. 2013.
23. Indirectness: We downgraded once since the study research question was not in line with the PICO question. In Shapiro et al. women received different types of ART depending on CD4 cell count whilst in Thomas et al. 2011 apart from all mothers being on ART all infants received a single dose of NVP within 72 hours and TMP/SMX from 6 weeks.
